# An examination of core beliefs in generalized anxiety disorder

**DOI:** 10.1111/bjc.70047

**Published:** 2026-03-25

**Authors:** Amy E. Doust, Vesna Trenoska Basile, Sarah McDonald, Toby Newton‐John, Bethany M. Wootton

**Affiliations:** ^1^ Discipline of Clinical Psychology, Graduate School of Health University of Technology Sydney Ultimo New South Wales Australia

**Keywords:** cognitive behavioural therapy, core beliefs, generalized anxiety disorder

## Abstract

**Objective:**

Generalised anxiety disorder (GAD) is characterised by uncontrollable worries. Cognitive behaviour therapy (CBT) is an effective treatment for GAD; however, manualised treatments rarely include interventions to target core beliefs. This study aimed to (1) explore the core beliefs about the self that are endorsed by individuals with GAD, (2) determine whether these beliefs are related to the severity of GAD symptoms, (3) examine if core beliefs about the self shift in response to CBT for GAD and (4) determine whether changes in core beliefs about the self are related to reductions in symptom severity.

**Method:**

A secondary data analysis of a randomised controlled trial with a repeated measures design examining the efficacy of videoconferencing‐delivered CBT for individuals with GAD. Seventy‐seven participants were included in the study (*M*_age =_ 37.14; 84.4% female).

**Results:**

The results indicated that core beliefs about the self were commonly endorsed in GAD; however, there was no significant relationship between core beliefs about the self and baseline GAD symptom severity. There was a significant reduction in the severity of core beliefs about the self from pre‐treatment to post‐treatment (*p* < .001; Cohen's *d =* .50) and pre‐treatment to follow‐up (*p* < .001; Cohen's *d =* .51) despite the treatment not explicitly targeting these beliefs. There was also a significant relationship between changes in core beliefs about the self and GAD symptom severity at post‐treatment and follow‐up.

**Conclusions:**

These findings provide insight into the role of core beliefs about the self in the treatment of GAD.

**Practitioner points**

- A number of negative core beliefs about the self were commonly endorsed by the sample, including: “*I am a weird person*”, “*I am inadequate*”, “*I am unlikable*”, and “*I am a weak person*”.
- There was no significant relationship between the presence of negative core beliefs about the self and the severity of GAD symptoms at baseline.
- Core beliefs about the self can shift in a standard CBT intervention that does not explicitly target them.
- There is a relationship between decreases in core beliefs about the self and decreases in symptom severity in GAD.
- Changes in core belief about the self predicted GAD symptom improvement at post‐treatment, but results were inconsistent at follow up.

## INTRODUCTION

Generalised anxiety disorder (GAD) is characterised by uncontrollable worries about multiple topics that are accompanied by physiological symptoms such as restlessness, fatigue, difficulty concentrating, muscle tension, sleep disturbances and irritability (American Psychiatric Association, 2022). GAD has a lifetime prevalence rate of 3.7% and a 12‐month prevalence of 1.8% (Ruscio et al., 2017), and women are more likely to receive a diagnosis of GAD compared to men (Australian Bureau of Statistics, 2021; Kessler et al., 2012). The disorder results in considerable psychosocial impairment (Ruscio et al., 2017) and is characterised by significant levels of comorbidity (Ruscio et al., 2017).

Cognitive behavioural therapy (CBT) is widely recognised as an effective treatment for GAD with large meta‐analyses demonstrating that CBT produces significant reductions in GAD symptoms compared to control conditions, with effect sizes ranging from medium to large (Carpenter et al., 2018; Cuijpers et al., 2014). A recent meta‐analysis found that CBT is durable, with sustained improvements in GAD symptoms six months after treatment (Carpenter et al., 2018). CBT is versatile in its delivery and can be administered effectively both in person (Stefan et al., 2019) and remotely via videoconferencing (Bouchard et al., 2022; Stubbings et al., 2013; Basile et al., 2024). Remote delivery of CBT for GAD can assist in overcoming barriers to accessing treatment (Trenoska Basile, Newton‐John, & Wootton, 2024).

Previous research has highlighted that individuals with GAD are likely to present with a range of cognitive distortions, including catastrophising, black or white thinking, mental filter and overgeneralisation (Özdemir & Kuru, 2023). However, few studies have examined deeper, more enduring cognitive structures, such as core beliefs. Core beliefs are fundamental convictions that shape one's self‐perception, perceptions of others and understanding of the world (Padesky, 1994) and are an important component of schemas (Young et al., 2003). In CBT, challenging core beliefs is considered to be a critical therapeutic strategy applied across various disorders in order to sustain treatment improvements. However, research on the specific impact of core beliefs on the development and maintenance of GAD remains limited. If core beliefs indeed influence the cognitive profiles of individuals with GAD, integrating this understanding into the current conceptualisation and treatment of the disorder could provide meaningful advancements in GAD outcomes.

To date, three studies have examined the relationship between core beliefs and GAD symptoms in non‐clinical and clinical samples. Firstly, Koerner et al. (2015) examined the role of core beliefs in developing and maintaining GAD beyond traditional factors like worry, uncertainty and problem‐solving. In this study, 138 participants were classified into ‘probable GAD’ and ‘non‐GAD’ groups. The strongest predictor of GAD group membership was perfectionism, including unrelenting standards and self‐sacrificial beliefs, followed by positive beliefs about worry, positive beliefs about others, depressive symptoms and negative beliefs about uncertainty (Koerner et al., 2015). Notably, two core beliefs uniquely predicted the presence of probable GAD or greater GAD severity after controlling for other belief sets, trait anxiety and depressive symptoms: (1) beliefs of unrelenting self‐standards or self‐sacrifice and (2) less positive views of others and their intentions (Koerner et al., 2015).

In studies using clinical samples, Kocakaya and Özturan (2023) also compared the core beliefs of individuals with GAD and non‐GAD controls, finding that GAD patients exhibited significantly higher scores in beliefs such as emotional deprivation, pessimism, approval seeking, self‐sacrifice, punitiveness, abandonment and vulnerability to harm/illness. Additionally, there was a significant positive correlation between GAD severity and the presence of these core beliefs (Kocakaya & Özturan, 2023). In a similar study, Popa et al. (2017) examined core beliefs in a clinical sample of GAD patients (*N* = 100) compared to a control group of 100 individuals without any psychiatric diagnoses. The study revealed that scores were significantly elevated in the GAD group compared to the control group on all of the schemas measured by the Young Schema Questionnaire Short Form Version 3 (YSQ‐S3; Young & Brown, 2005) (Popa et al., 2017).

In summary, whilst targeting core beliefs is a widely employed strategy in CBT, empirical evidence identifying the specific core beliefs associated with GAD and their influence on treatment responsivity remains scarce. Therefore, the primary aim of the current study is to investigate core beliefs in adults diagnosed with GAD. Specifically, this research seeks to (1) explore the specific core beliefs that individuals with GAD endorse, (2) determine whether these beliefs are related to the severity of GAD symptoms at baseline, (3) determine whether these beliefs shift after standard CBT for GAD (i.e., CBT that does not explicitly target core beliefs) and (4) determine whether changes in core beliefs are related to change in symptom severity after CBT. Given the limited research in this field, the study was designed as exploratory with no a priori hypotheses. This study seeks to fill a critical research gap and aims to enhance treatment outcomes for patients with GAD.

## METHODS

### Design

The present study employed a secondary data analysis of a randomised controlled trial that examined the efficacy of videoconferencing‐delivered CBT for GAD (Basile et al., 2024) In this study, participants were randomised to either 8 sessions of CBT for GAD or a waitlist control group. The control group was provided the same treatment in a brief format (i.e., 5‐sessions) after they finished serving as the control group. In this study, treatment scores were combined for both groups, given that the treatments resulted in similar outcomes (Trenoska Basile, Newton‐John, & Wootton, 2024). The data included in the current study include the pre‐treatment, post‐treatment (8‐weeks after pre‐treatment) and 3‐month follow up for the immediate treatment group and pre‐treatment, post‐treatment (5‐weeks after pre‐treatment) and 3‐month follow up for the waitlist control group after they had served as the waitlist (i.e., when the participants were in the treatment phase).

### Participants

Seventy‐eight participants were included in the original study; however, one control group participant did not complete the baseline questionnaires, and thus, 77 participants were included in the current study. On average, participants were in their mid to late 30s (*M* = 37.14; *SD* = 12.92). The majority of the sample was female (65/77; 84.4%), with smaller proportions identifying as male (10/77; 13.0%) and non‐binary/gender diverse (2/77; 2.6%). Twenty‐four out of the 77 participants (31.2%) were taking medication related to their symptoms. Common comorbidities included major depressive disorder (39/77; 50.6%), social anxiety disorder (33/77; 42.9%), and obsessive‐compulsive disorder (21/77; 27.3%). The demographic characteristics of the sample are outlined in Table 1.

**TABLE 1:** Participant characteristics (*N* = 77).

Variable	*n*	*%*
Gender		
Female	65	84.4
Male	10	13.0
Non‐binary/gender diverse	2	2.6
Age		
Mean (*SD*)	37.14 (12.92)	–
Range	18–65	–
Marital status		
Single	35	45.5
Married/de facto	34	44.2
Divorced/separated	5	6.5
Other	3	3.9
Education		
High school	21	27.3
Trade certificate/diploma	11	14.3
Bachelor degree	26	33.8
Master/doctoral degree	19	24.7
Medication (% yes)	24	31.2
Comorbid diagnoses		
Major depressive disorder	39	50.6
Social anxiety disorder	33	42.9
Obsessive compulsive disorder	21	27.3
Panic disorder	14	18.2
Persistent depressive disorder	14	18.2
Attention deficit/hyperactivity disorder	14	18.2
Specific phobia	12	15.6
Premenstrual dysphoric disorder	11	14.3
Agoraphobia	10	13.0
Body dysmorphic disorder	9	11.7
Separation anxiety disorder	8	10.4
Hoarding disorder	7	9.1
Excoriation disorder	7	9.1
Substance use disorder	7	9.1
Adjustment disorder	6	7.8
Somatic symptom disorder	5	6.5
Illness anxiety disorder	4	5.2
Posttraumatic stress disorder	3	3.9
Binge eating disorder	2	2.6
Bipolar II disorder	1	1.3
Tic disorder	1	1.3
GAD‐7 total score	12.53 (4.10)	–
GAD‐D total score	18.58 (7.10)	–
CBQ total score	55.74 (17.95)	–
PHQ‐9 total score	11.44 (4.73)	–

To be included in the original study, participants were required to reside in Australia, be 18 years or older, be fluent in English and have a primary diagnosis of GAD with at least moderate severity, indicated by a score of 4 on the Diagnostic Interview for Anxiety, Mood, and OCD and Related Neuropsychiatric Disorders (DIAMOND; Tolin et al., 2018). Participants also needed to be medication‐free or on a stable dose of psychotropic medication and not be receiving regular (i.e., at least weekly) psychological therapy for their GAD symptoms.

Exclusion criteria included severe depressive symptoms, defined by a score of 20 or above on the Patient Health Questionnaire‐9 (PHQ‐9; Kroenke et al., 2001), or suicide risk, identified by a score of 2 or higher on item 9 of the PHQ‐9 (Kroenke et al., 2001) or through clinician judgment using the Columbia‐Suicide Severity Rating Scale (Posner et al., 2011). Daily use of alcohol or illicit drugs, a schizophrenia spectrum disorder, significant cognitive or intellectual impairment, medical conditions interfering with treatment or lack of access to a computer with a camera and a stable internet connection also led to exclusion. Further details about study flow and screening measures can be found in the published protocol (Trenoska Basile et al., 2022).

### Measures

#### Demographics

Participants provided details on their age, geographical location, gender, marital status, employment status, educational background, medication usage and other relevant demographic details.

#### Core Beliefs Questionnaire (CBQ) ‐ trait version (Wong et al., 2017)

The CBQ Trait Version is a 17‐item measure of core beliefs about the self. Participants rated their agreement with each statement (e.g., “I am unlikeable”) on a 6‐point Likert scale, ranging from 1 (strongly disbelieve) to 6 (strongly believe). Higher scores reflected a stronger endorsement of negative core beliefs about oneself. The CBQ exhibits strong psychometric properties, including a single‐factor structure, excellent internal consistency and the ability to differentiate between individuals with and without social anxiety disorder (Wong et al., 2017) and with and without GAD (Kuru et al., 2025). The CBQ scores are positively associated with social anxiety symptoms, and reductions in these scores from pre‐treatment to post‐treatment predict treatment outcomes (Wong et al., 2017). In this study, a participant was considered to endorse a core belief if they scored a ‘4’, ‘5’ or ‘6’ on the relevant item. The internal consistency in the current study was .94.

#### Generalised Anxiety Disorder Questionnaire – 7 item (GAD‐7; Spitzer et al., 2006)

The GAD‐7 is a 7‐item self‐report instrument that assesses the symptoms of GAD. Each item is rated on a scale from 0 (not at all) to 3 (nearly every day). The GAD‐7 demonstrates strong psychometric properties, including high internal consistency, good test–retest reliability and effective unidimensional measurement of GAD (Hinz et al., 2017; Spitzer et al., 2006). The GAD‐7 also shows robust concurrent and diagnostic validity, correlating well with other anxiety measures and accurately identifying cases of GAD (Hinz et al., 2017). A cumulative score of 10 or above indicated clinically significant symptoms of GAD (Hinz et al., 2017; Spitzer et al., 2006). The internal consistency in the current study was .81.

#### Generalised Anxiety Disorder Dimensional Scale (GAD‐D; Lebeau et al., 2012)

The GAD‐D is a 10‐item measure of generalised anxiety symptoms. Participants rate the frequency with which they have experienced GAD symptoms over the past month on a 5‐point Likert scale ranging from 0 (Never) to 4 (All of the time), resulting in a total score ranging between 0 and 40. The GAD‐D demonstrates excellent psychometric properties in clinical and non‐clinical samples (Beesdo‐Baum et al., 2012; Lebeau et al., 2012) including in Australia (Groves et al., 2023). The internal consistency in the current study was .86.

#### Patient Health Questionnaire – 9 item (PHQ‐9; Kroenke et al., 2001)

The PHQ‐9 is a commonly used nine item measure of depressive symptoms. Symptoms are assessed over the previous two weeks. Participants rate each question on a 0 (*not at all*) to 3 (*nearly every day*) scale. Total scores ≥10 indicate clinically significant depressive symptoms (Manea et al., 2012). The PHQ‐9 has been demonstrated to have excellent psychometric properties in previous samples (Kroenke et al., 2001; Zuithoff et al., 2010). The internal consistency in the current study was .77.

### Procedure

Ethical approval for the wider study was obtained from the University of Technology Sydney Health and Medical Research Ethics Committee (REF NO. ETH21‐5843). The original study was pre‐registered with the Australian and New Zealand Clinical Trial Registry (ACTRN12621000786897). Approval for the secondary data analysis reported in this study was also obtained from the University of Technology Sydney Health and Medical Research Ethics Committee (ETH23‐8647).

Participants were recruited via the study website, social media ads, emails to clinicians, flyers and not‐for‐profit organisations. Through a REDCAP survey link, they reviewed consent, completed assessments and provided contact details. A telephone interview confirmed GAD diagnosis and comorbidities using the DIAMOND (Tolin et al., 2018). Eligible participants completed online questionnaires at baseline, mid‐treatment, post‐treatment and three‐month follow‐up. Non‐eligible participants were referred to support services.

### Treatment

The treatment delivered in the original study (Basile et al.,  2024, 2025) followed a manualised protocol that was based on the CBT manual of Dugas and Robichaud (2007) and Robichaud et al. (2019). This treatment manual comprised six modules including: (1) psychoeducation, (2) cognitive restructuring, (3) behavioural experiments, (4) problem‐solving training, (5) imaginal exposure and (6) relapse prevention. Participants assigned to the immediate treatment group received 10 weekly sessions (50 min each). The control group received a condensed five‐week version of the same intervention, with one session per week (50 min each) and similar coverage of the six modules, albeit with reduced time allocated to each component. Treatments were delivered individually and via the internet‐videoconferencing program Zoom. There was no explicit targeting of core beliefs in the treatment.

### Data analysis

Data was analysed using SPSS Version 28.0. To examine the specific core beliefs that are endorsed by individuals with GAD, descriptive statistics were used. To determine whether core beliefs are related to the severity of GAD symptoms, the CBQ total score was correlated with the GAD‐7 and GAD‐D. We also examined the correlation between the CBQ and the PHQ‐9. We examined whether each core belief was correlated with the GAD‐7 and GAD‐D, and in these analyses, we controlled for baseline PHQ‐9 scores.

Mixed linear models were used to examine change in the CBQ scores in the treatment and waitlist control group, and between‐group effect sizes were calculated at post‐treatment. We also assess change on the CBQ from pre‐treatment to post‐treatment and pre‐treatment to 3‐month follow up. For these analyses, scores for all participants who completed the treatment were combined (i.e., the waitlist control after they had completed the treatment), and within‐group effect sizes were calculated from pre‐treatment to post‐treatment and pre‐treatment to 3‐month follow up. All within‐group and between‐group effect sizes are reported as Cohen's *d* with 95% confidence intervals and were interpreted as *d* = .20 (small effect) *d* = .50 (medium effect) and *d* = .80 (large effect) (Cohen, 1988).

To determine whether changes in core beliefs are related to changes in symptom severity after CBT, a ‘change score’ was first calculated for each participant on the GAD‐7, GAD‐D and CBQ. The change score was calculated by subtracting the post‐treatment (or follow‐up) total scores from the pre‐treatment total scores and dividing by the pre‐treatment total on each measure. Correlations were then conducted between the CBQ change score and each of the symptom severity change scores (GAD‐7 and GAD‐D) at post‐treatment and follow‐up. For correlational analyses, the Cohen (1988) convention was used to define the effect size strength of the correlation coefficients (small: *r* = .10 to .29; moderate: *r* = .30 to .49; strong: *r* ≥ .50).

Finally, regression analyses were conducted to examine whether a change in scores on the CBQ at post‐treatment predicted GAD symptom improvement on the GAD‐7 and GAD‐D at post‐treatment and follow up. For these analyses, we conducted the linear regressions both with and without controlling for depressive symptoms. In these analyses, the *f* ^2^ statistic is used as the effect size, and these were interpreted as .02 (small), .15 (medium), and > .35 (large) (Cohen, 1992).

## RESULTS

### Core beliefs about the self endorsed by individuals with generalized anxiety disorder

The mean scores on each item of the CBQ, as well as the percent of participants endorsing each of the core beliefs in the CBQ, is outlined in Table 2. The core beliefs about the self with the highest mean scores were “*I am inadequate*” (*M =* 3.95; *SD* = 1.49), “*I am a weird person*” (*M =* 3.84; *SD* = 1.50), “*I am physically unattractive*” (*M =* 3.75; *SD* = 1.41), “*I am a failure*” (*M =* 3.52; *SD* = 1.54) and “*I am uninteresting*” (*M =* 3.47; *SD* = 1.52). The most frequently endorsed core beliefs about the self were “*I am a weird person*” (endorsed by 67.5% of the sample), “*I am inadequate*” (endorsed by 66.2% of the sample), “*I am unlikeable*” (endorsed by 57.1% of the sample), “*I am a weak person*” (endorsed by 57.1% of the sample) and “*I am physically unattractive*” (endorsed by 55.8% of the sample).

**TABLE 2:** Item‐by‐item descriptive statistics for the core beliefs questionnaire (*N* = 77).

Variable	*M*	*SD*	Min	Max	*N*	%
CBQ total	55.74	17.96	17	97	–	–
“I am unlikeable”	3.43	1.34	1	6	44	57.1
“I am foolish”	3.31	1.40	1	6	40	51.9
“I am inadequate”	3.95	1.49	1	6	51	66.2
“I am inferior”	3.39	1.57	1	6	39	50.6
“I am uninteresting”	3.47	1.52	1	6	40	51.9
“I am dumb/stupid”	2.58	1.39	1	6	24	31.2
“I am a weak person”	3.45	1.60	1	6	44	57.1
“I am incompetent”	3.07	1.51	1	6	34	44.2
“I am unacceptable”	2.68	1.45	1	6	23	29.9
“I am not a worthwhile person”	2.84	1.51	1	6	25	32.5
“I am a weird person”	3.84	1.50	1	6	53	67.5
“I am unimportant”	3.27	1.47	1	6	37	48.1
“I am physically unattractive”	3.75	1.41	1	6	43	55.8
“I am inept”	2.96	1.52	1	6	31	40.3
“I am unlovable”	3.01	1.44	1	6	28	36.4
“I am a failure”	3.52	1.54	1	6	42	54.5
“I am defective”	3.21	1.41	1	6	37	48.1

*Note*: The percentage outlines the participants who endorsed a ‘4’, ‘5’ or ‘6’ on each core belief.

### Relationship between GAD symptom severity and core belief endorsement

There were no significant correlations between the total score on the CBQ and the GAD‐7 (.093, *p* > .05) or GAD‐D (.141, *p* > .05) at baseline. There was a significant correlation between the CBQ and PHQ‐9 (.287, *p* = .012) at baseline. The correlations between each item on the CBQ and the GAD‐7 and GAD‐D, when controlling for baseline depression scores, are outlined in Table 3. None of these correlations was significant.

**TABLE 3:** Correlation coefficients between GAD‐7 and GAD‐D total scores at baseline and individual items on the CBQ when controlling for depression (*N* = 77).

Core belief questionnaire	GAD‐7	GAD‐D
“I am unlikeable”	−.226	−.160
“I am foolish”	−.038	−.112
“I am inadequate”	−.003	−.104
“I am inferior”	.027	−.102
“I am uninteresting”	−.178	−.098
“I am dumb/stupid”	−.060	−.014
“I am a weak person”	.100	.081
“I am incompetent”	.095	.062
“I am unacceptable”	.037	.007
“I am not a worthwhile person”	−.008	−.144
“I am a weird person”	−.168	−.113
“I am unimportant”	−.057	−.064
“I am physically unattractive”	−.200	−.121
“I am inept”	.102	.074
“I am unlovable”	.075	.044
“I am a failure”	.013	.020
“I am defective”	.032	.025

### Changes in core beliefs about the self after cognitive behaviour therapy

#### Treatment vs. control

Pre‐treatment and post‐treatment estimated marginal means and standard deviations for the treatment and control groups on the CBQ are outlined in Table 4. There was a significant effect for Group (*F*_(1, 137)_ = 5.265, *p* = .023) and Time (*F*_(1,137)_ = 4.221, *p* = .042) and but not for the Group by Time interaction (*F*_(1, 137)_ = 2.040, *p* = .155). Pairwise comparisons revealed that there was a significant change on the CBQ from pre‐treatment to post‐treatment in the treatment group (*d* = 0.55, 95% *CI*: 0.09–1.00), but not the control group (*d* = 0.10, 95% *CI*: −0.35 – 0.55). The between‐group effect size at pre‐treatment was non‐significant (*d =* 0.15; 95% *CI*: −0.30 *–* 0.60), and the between‐group effect size at post‐treatment was medium *(d =* 0.58; 95% *CI*: 0.12–1.03).

**TABLE 4:** Estimated marginal means and standard deviations on the CBQ for treatment (*N* = 38) and control (*N* = 38) at pre‐treatment and post‐treatment.

	Pre‐treatment	Post‐treatment	Within‐group effect size (Cohen's *d*) and 95% CI	Between‐group effect size at post‐treatment (Cohen's *d*) and 95% CI
Treatment	54.66 (18.64)	43.66 (21.34)	0.55 (0.09–1.00)	0.58 (0.12–1.03)
Control	57.4 (18.64)	55.42 (19.15)	0.10 (−0.35–0.55)	

#### Change over time

The estimated marginal means and standard deviations at pre‐treatment, post‐treatment and 3‐month follow‐up on the CBQ for all participants after they commenced treatment, as well as effect sizes with 95% confidence intervals, are outlined in Table 5. The analyses indicated a significant effect for Time on the CBQ (*F*_2,94.91_ = 16.085, *p* < .001). Pairwise comparisons indicated a significant change on the CBQ from pre‐treatment to post‐treatment (*p* < .001; *d =* .50, 95% CI: 0.17–0.82) and from pre‐treatment to 3‐month follow‐up (*p* < .001; *d =* .51, 95% CI: 0.19–0.84), both with a medium effect size. There was no significant change from post‐treatment to 3‐month follow‐up.

**TABLE 5:** Estimated marginal means and standard deviations on the CBQ for all participants at pre‐treatment, post‐treatment and follow up (*N* = 75).

	Pre‐treatment	Post‐treatment	Follow up	Within‐group effect size pre‐treatment to post‐treatment (Cohen's *d*) and 95% CI	Within‐group effect size pre‐treatment to follow up (Cohen's *d*) and 95% CI
Treatment	54.75 (19.45)	45.158 (19.17)	44.307 (21.13)	.50 (0.17–0.82)	.51 (0.19–0.84)

### Are changes in core beliefs about the self related to change in symptom severity?

The correlation between the CBQ change score and the GAD‐7 change score was significant at post‐treatment (*r* = .288, *p* = .035, medium effect) and follow‐up (*r* = .451, *p* < .001, medium effect). A similar pattern emerged for the GAD‐D, where the correlation between the CBQ change score and GAD‐D change score was significant at post‐treatment (*r* = .379, *p* < .01, medium effect) and follow‐up (*r* = .434, *p* = .001, medium effect).

Regression analyses were conducted to examine whether a change in scores on the CBQ at post‐treatment predicted GAD symptom improvement on the GAD‐7 and GAD‐D at post‐treatment and follow up. For the GAD‐7 at post‐treatment, the regression was significant (*F*_(1,53)_ = 4.707, *p* = .035) with approximately 8% of the variance explained by change in CBQ scores (*R* ^2^ = .08; Adjusted *R* ^2 =^ .065), representing a small effect size (*f* ^2^ = 0.09). The results were consistent when controlling for baseline depressive symptoms (*F*_(2,53)_ = 3.673, *p* = .03; *R* ^2^ = .126; Adjusted *R* ^2 =^ .092). At follow up, the regression was no longer significant (*F*_(1,49)_ = 3.694, *p* = .061).

For the GAD‐D, the regression was also significant at post‐treatment (*F*_(1,53)_ = 8.696, p < .01) with approximately 14% of the variance explained by change in CBQ scores (*R* ^2^ = .143; Adjusted *R* ^2 =^ .127), representing a medium effect size (*f* ^2^ = 0.16). The results were consistent when controlling for baseline depression symptoms (*F*_(2,53)_ = 6.394, *p* = .003; *R* ^2^ = .20; Adjusted *R* ^2 =^ .169). At follow up the regression remained significant (*F*_(1,50)_ = 5.419, *p* = .024) with approximately 10% of the variance in follow up scores explained by the change in CBQ scores from pre‐treatment to post‐treatment (*R* ^2^ = .10; Adjusted *R* ^2 =^ .0.81), representing a small effect size (*f* ^2^ = 0.11). These results were maintained even when controlling for baseline depressive symptoms (*F*_(2,50)_ = 4.705, *p* = .014).

## DISCUSSION

The present study aimed to investigate core beliefs about the self in adults diagnosed with GAD. Specifically, this paper aimed to (1) explore the specific core beliefs about the self that individuals with GAD endorse, (2) determine whether these beliefs are related to the severity of GAD symptoms at baseline, (3) whether these beliefs shift after standard CBT for GAD and (4) determine whether changes in core beliefs about the self are related to change in symptom severity after CBT. The study was designed as exploratory with no a priori hypotheses.

When examining the core beliefs about the self, the items of the CBQ with the highest mean scores were “*I am inadequate*”, “*I am a weird person*”, “*I am physically unattractive*”, “*I am a failure*” and “*I am uninteresting*”. The core beliefs about the self that were most frequently endorsed by individuals with GAD were “*I am a weird person*”, “*I am inadequate*”, “*I am unlikable*” and “*I am a weak person*”. These results suggest that negative core beliefs about the self are common in individuals with GAD; however, it is unclear if this is related to GAD specifically, or mental health disorders more broadly, and further research is required.

It is difficult to compare the current findings with the existing literature, given that previous studies have used different measures to assess core beliefs. For example, the endorsement of beliefs such as “*I am inadequate*” aligns with the work of Koerner et al. (2015), who found that perfectionism and beliefs relating to unrelating self‐standards predicted the presence of likely GAD in those without GAD. Similarly, the identification of beliefs such as “*I am a weird person*”, “*I am unlikable*” and “*I am a weak person*” is consistent with the findings of Popa et al. (2017) who found that individuals with GAD had elevated scores on all of the schemas measured by the Young Schema Questionnaire Short Form Version 3 (YSQ‐S3; Young & Brown, 2005) (Popa et al., 2017). However, it is important to examine in future research if these beliefs are triggered by an episode of worry or are more global. If triggered by an episode of worry they may be better explained as negative metacognitive beliefs consistent with the metacognitive model of worry (MCM; Wells, 1995). To advance this literature further, it is important for future research to compare the core belief profiles amongst individuals with different mental health disorders using the same measure of core beliefs, and these should be compared with a non‐clinical control group. It is also important to further examine when these beliefs are triggered, specifically, whether they are triggered by an episode of worry or are more ingrained and triggered in other contexts.

The present study also found that there was no significant relationship between total scores on the CBQ and either of the GAD symptom measures. When controlling for baseline depressive symptoms, none of the core beliefs correlated with the GAD symptom measures. This finding is consistent with a recent study examining the relationship between the CBQ total score and measures of social anxiety symptoms (Winter et al., 2025) but inconsistent with a similar study that found a significant positive relationship between the CBQ total score and a measure of social anxiety symptoms, with a large effect (Wong et al., 2017). Similarly, Kocakaya and Özturan (2023) found a significant positive correlation between GAD severity and the presence of maladaptive schemas using the Young Schema Questionnaire Short Form Version 3 (YSQ‐S3; Young & Brown, 2005). While further research is needed, this finding indicates that while problematic core beliefs about the self may be present in individuals with GAD, they may not be directly related to GAD symptom severity.

While baseline GAD severity was not related to the severity of core beliefs about the self at baseline, this study did find a significant relationship between change in GAD severity and change in core beliefs about the self as measured by the CBQ at both post‐treatment and 3‐month follow‐up. Regression analyses indicated that changes in the CBQ significantly predicted changes in GAD symptoms at post‐treatment on both symptom measures with small to medium effects (i.e., the GAD‐7 and GAD‐D); however, at follow up, the regression was no longer significant for the GAD‐7, but was on the GAD‐D. While further research is needed, it is possible that while core beliefs about the self are not related to GAD severity at baseline, the shift in these core beliefs may contribute to increased responsiveness to treatment. Thus, while core beliefs seem to change without directly addressing them in CBT for GAD, it is possible that increased benefit may be obtained from explicitly addressing them, at least for some patients. It is important for future studies to examine this research question.

The CBT intervention delivered in this study did not explicitly target core beliefs. However, despite this, the results indicate that there was a significant change in core beliefs about the self from pre‐treatment to post‐treatment and from pre‐treatment to 3‐month follow‐up in the immediate treatment group. The change in the control group was non‐significant. The strength of this change was medium, suggesting that it may be clinically meaningful; however, further research is required. While to our knowledge this is the first study to examine change in core beliefs about the self during CBT for GAD, these findings are consistent with research in other disorders. For example, previous research in a SAD sample also found that the CBQ is responsive to change after CBT (Winter et al., 2025; Wong et al., 2017) and that decreases in the severity of core beliefs were associated with decreases in the severity of social anxiety symptoms (Wong et al., 2017). However, in this study, two of the 12 sessions were devoted to addressing problematic core beliefs (Wong et al., 2017). Similarly, Cruwys et al. (2014) found across two studies that social isolation schemas reduced significantly in a group CBT treatment program for depression and anxiety without specifically addressing it, but also that social isolation schemas reduced in a non‐therapeutic group setting of homeless individuals residing in temporary accommodation.

These results can be interpreted in a number of ways. Firstly, if core beliefs about the self reduce through standard cognitive–behavioural interventions, there may be no need to target them explicitly in treatment given this will add to the number of treatment sessions required, and patients commonly report that cost of treatment is a major barrier to accessing treatment for GAD (Trenoska Basile, Newton‐John, & Wootton, 2024), and less sessions makes the treatment more affordable. Secondly, and alternatively, these results may indicate that while core beliefs about the self can shift in standard CBT for GAD, they may shift further when core beliefs are directly targeted in treatment. Finally, the results of Cruwys et al. (2014) indicate that core beliefs can shift in individuals who are not engaged in a therapeutic treatment, but have a group based social experience and changes may be due to non‐specific factors. It will be important to examine in future research if explicitly targeting core beliefs in a standard CBT for GAD protocol enhances treatment effectiveness or whether direct targeting or core beliefs is not needed in the treatment of GAD. Such research would benefit from regular tracking (i.e., weekly) of changes in core beliefs alongside changes in GAD symptoms to see if there is a relationship between these variables.

### Limitations

While this study is an important addition to the literature examining the role of core beliefs about the self in GAD, there are also a number of limitations that should be acknowledged. Firstly, this study did not include a control group of individuals with other mental health disorders. It would be valuable to replicate the study using a control group with a different anxiety disorder, as well as a non‐clinical control group. Such research may also include a group of individuals with a primary depressive disorder to ascertain if core beliefs about the self are qualitatively different from those seen in anxiety disorders.

Secondly, this study may be limited by the tool used to assess core beliefs. The CBQ is a more recently developed measure of core beliefs about the self, and while the psychometric properties of the tool are strong (Wong et al., 2017), future research may wish to replicate our findings using other measures of core beliefs, especially those that examine core beliefs more generally, as opposed to core beliefs about the self, which was specifically measured in this study.

Thirdly, this study was a secondary data analysis, and to increase power for some analyses, we combined participants who completed two different treatments into one group. While both groups received the same treatment, one group received 10 sessions (Basile et al., 2024), while the other received 5 sessions (Basile et al., 2025). Outcomes in GAD symptoms were similar across both groups (Basile et al., 2025), but the difference in the number of sessions may have introduced statistical ‘noise’ into our analyses. Nonetheless, the current research highlights the need for future research to further examine the role of core beliefs a priori, with a larger sample size.

Finally, the sample was primarily female, and there were very few individuals identifying as non‐binary or gender diverse. As such, these results may not be generalisable to other genders. Additionally, the sample was highly educated with more than half the same obtaining a tertiary qualification. To address this limitation, future research should endeavour to recruit a more diverse sample. The sample was however diverse in terms of comorbid conditions. While high levels of comorbidity are common in GAD (Ruscio et al., 2017), the results may be impacted by these comorbidities, and future research may wish to examine these research questions in a more homogenous sample in terms of clinical presentations.

## CONCLUSION

The aim of this study was to assess core beliefs about the self in a sample of patients with primary GAD. The study found that while core beliefs about the self may be present in individuals with GAD, they are not necessarily related to GAD severity at baseline. However, there may be a relationship between a change in core beliefs and a change in symptom severity. Additionally, the study found that core beliefs may shift in a clinically meaningful way during a standard CBT intervention that does not explicitly target them. Further research is required to replicate our findings and elucidate whether core beliefs may be more or less important clinical targets when compared to other mental health disorders.

## AUTHOR CONTRIBUTIONS

**Amy E. Doust:** Formal analysis; visualization; writing – original draft. **Vesna Trenoska Basile:** Methodology; project administration; writing – review and editing. **Sarah McDonald:** Supervision; writing – review and editing. **Toby Newton‐John:** Supervision; writing – review and editing. **Bethany M. Wootton:** Conceptualization; methodology; formal analysis; supervision; visualization; writing – original draft; writing – review and editing.

## FUNDING INFORMATION

Vesna Trenoska Basile was supported by a Research Training Program scholarship from the Australian Government.

## CONFLICT OF INTEREST STATEMENT

The authors report there are no competing interests to declare.

## ETHICS STATEMENT

The original study was pre‐registered with the Australian and New Zealand Clinical Trials registry (ACTRN12621000786897).

## Data Availability

The data of the present study are available upon request from the corresponding author.
